# Optimizing Stroke Prevention in Patients With Atrial Fibrillation: A Cluster-Randomized Controlled Trial of a Computerized Antithrombotic Risk Assessment Tool in Australian General Practice, 2012–2013

**DOI:** 10.5888/pcd13.160078

**Published:** 2016-07-14

**Authors:** Beata V. Bajorek, Parker J. Magin, Sarah N. Hilmer, Ines Krass

**Affiliations:** Author Affiliations: Parker Magin, University of Newcastle, Callaghan, New South Wales, Australia; Sarah Hilmer, Royal North Shore Hospital, St Leonards, New South Wales, Australia; Ines Krass, University of Sydney, New South Wales, Australia. Dr Bajorek is also affiliated with Royal North Shore Hospital, St Leonards, New South Wales, Australia.

## Abstract

**Introduction:**

Clinicians have expressed a need for tools to assist in selecting treatments for stroke prevention in patients with atrial fibrillation. The objective of this study was to evaluate the impact of a computerized antithrombotic risk assessment tool (CARAT) on general practitioners’ prescribing of antithrombotics for patients with atrial fibrillation.

**Methods:**

A prospective, cluster-randomized controlled trial was conducted in 4 regions (in rural and urban settings) of general practice in New South Wales, Australia (January 2012–June 2013). General practitioner practices were assigned to an intervention arm (CARAT) or control arm (usual care). Antithrombotic therapy prescribing was assessed before and after application of CARAT.

**Results:**

Overall, the antithrombotic therapies for 393 patients were reviewed by 48 general practitioners; we found no significant baseline differences in use of antithrombotics between the control arm and intervention arm. Compared with control patients, intervention patients (n = 206) were 3.1 times more likely to be recommended warfarin therapy (over any other treatment option; *P* < .001) and 2.8 times more likely to be recommended any anticoagulant (in preference to antiplatelet; *P* = .02). General practitioners agreed with most (75.2%) CARAT recommendations; CARAT recommended that 75 (36.4%) patients change therapy. After application of CARAT, the proportion of patients receiving any antithrombotic therapy was unchanged from baseline (99.0%); however, anticoagulant use increased slightly (from 89.3% to 92.2%), and antiplatelet use decreased (from 9.7% to 6.8%).

**Conclusion:**

Tools such as CARAT can assist clinicians in selecting antithrombotic therapies, particularly in upgrading patients from antiplatelets to anticoagulants. However, the introduction of novel oral anticoagulants has complicated the decision-making process, and tools must evolve to weigh the risks and benefits of these new therapy options.

## Introduction

Treatment selection in the context of stroke prevention for people with atrial fibrillation (AFib) has become more complex (1,2). The increasing complexity is due in part to the challenges of treating the at-risk elderly population, in whom AFib is most prevalent and risk of stroke greatest (3,4), and to an expanded range of treatment options (ie, novel oral anticoagulants [NOACs]) used to remedy some of the difficulties of anticoagulation associated with warfarin (previously the mainstay therapy) (5). Although NOACs offer some advantages, they are not devoid of risk (6), and their high costs have given rise to recommendations for restricting their use and for better supporting people who use warfarin (7).

Overall, a greater range of factors must now be considered in weighing the risks and benefits of treatment, and this expanded range translates to a more complex decision-making process, which further contributes to the suboptimal use of antithrombotic therapy (8,9). This complexity adds to the burden of care in the general practice setting, where general practitioners (GPs) are principally responsible for decision making and day-to-day management (10,11). Although strategies and resources (eg, point-of-care testing, anticoagulation clinics, practice nurses, dedicated home-based warfarin management services) for supporting the management of anticoagulant therapy have been evaluated (12), less attention has been paid to decision-making processes. Simple scoring tools are available for GPs to assess the risks and benefits of treatment, particularly for stroke risk assessment (13), but tools to guide selection of treatment for patients are lacking (10,11).

A paper-based holistic risk-assessment algorithm for selecting treatment significantly improved the use of antithrombotic therapy (14). This algorithm was redeveloped into a Web-based computerized antithrombotic risk assessment tool (CARAT). CARAT facilitates a systematic review of risk factors and calculates the estimated risk-versus-benefit of therapy for each patient. Clinician feedback has suggested the potential utility of CARAT in general practice (15). The objective of this study was to evaluate the effect of CARAT on GPs’ prescribing of antithrombotic therapy. We also assessed changes made to patients’ therapy and the level of agreement on recommendations between GPs and CARAT.

## Methods

We evaluated the effect of CARAT in a prospective, cluster-randomized controlled clinical trial (trial registration no. ACTRN12613000060741); protocol details are provided elsewhere (16). Approval for the study was granted by the human research ethics committees of the University of Technology Sydney, University of Sydney, and University of Newcastle.

GPs were recruited from 4 regions of general practice in New South Wales, Australia: the northern suburbs of Sydney, the Central Coast, the Newcastle metropolitan area (in the Hunter Region), and the rural areas of the Hunter Region. These regions encompass rural and urban settings and have a high proportion of elderly patients who are diverse in socioeconomic status, health status, and access to health services.

**Figure 1:**
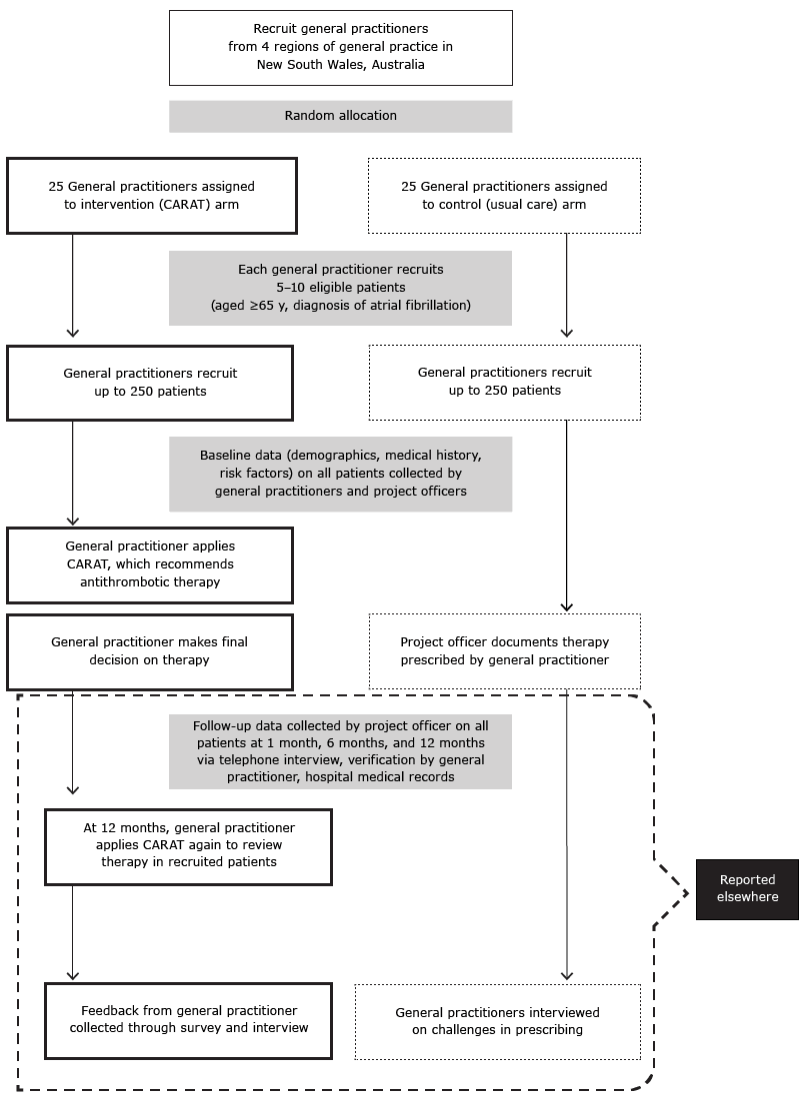
Schematic outline of a cluster-randomized controlled trial of a computerized antithrombotic risk assessment tool (CARAT) in a sample of general practices in New South Wales, Australia, 2012

GP practices were randomly allocated to one of 2 study arms, the intervention arm or the control arm (Figure 1). GPs in the control arm followed their usual practices; they used their own clinical judgment, processes, and resources when reviewing and selecting antithrombotic therapy for patients with AFib. GPs in the intervention arm used CARAT to support their decision-making processes.

CARAT assesses a patient’s stroke risk, bleeding risk, and medication-safety issues to quantify the patient’s estimated risk of stroke versus bleeding, flag medication-management issues, and generate a recommendation for therapy (warfarin, aspirin, none, or other) (15). GPs in the intervention arm were asked to consider CARAT output while reviewing the patient’s therapy and then make a final decision to initiate, cease, or maintain current therapy. When GPs disagreed with CARAT’s recommendations, the rationale for disagreement was documented in CARAT. During the study, not all NOACs were available or listed on the Australian Pharmaceutical Benefits Scheme and hence were not listed as treatment options by CARAT (except as “other” therapy); only dabigatran (Pradaxa) was available as part of the sponsoring company’s product familiarization program, which offered patients up to 12 months of therapy at no cost (17,18), 

Family (general) practice-based GPs who practiced at one site (ie, not at multiple sites) were recruited into the study from each region. They were randomly allocated through computer-generated random allocation number sequences and then asked to recruit patients during a 3-month period, from January through March 2012. Patients were selected for participation in the study if they were eligible according to the following criteria:

- Aged 65 years or older (ie, moderate to high risk of stroke by definition)
- Diagnosed with AFib, as confirmed by the GP (ie, pre-existing or new onset, regardless antithrombotic therapy prescribed)
- Able to provide informed written consent to participate

The target sample size was 25 GPs for each study arm, with each GP recruiting 5 to 10 patients (ie, up to 250 patients per study arm), as estimated from previous data (8,14). This sample size accounted for any clustering effects (a prior intracluster correlation = 0.03, 80% power) and a participant dropout rate of up to 20% (19,20).

### Data collection

A purpose-designed data collection form for classifying stroke risk and bleeding risk was used by GPs and project officers to document information on patient demographics, medical history (including concurrent chronic diseases defined as non-self-limiting conditions persisting for at least 3 months [21]), AFib, medication management and safety issues, contraindications to warfarin therapy, and antithrombotic therapy prescribed. The clinical information required to assess stroke risk, bleeding risk, medication adherence, potential for drug interactions, and medication-management support needs were inputs for CARAT. Patients were assessed for risk of stroke according to the CHADS_2_ Score for Atrial Fibrillation Stroke Risk (25) and for risk of bleeding according to HEMORR_2_HAGES criteria (26) and categorized as being at low, medium, or high risk. Factors affecting the decision to prescribe antithrombotic therapy (eg, ability of patient to manage therapy, relative contraindications to therapy) were documented for each patient.

### Data analysis

Computerized data analysis of patient outcomes was performed by using SPSS Statistics 20 (IBM Corp). Analysis of variance tested for mean differences in continuous variables, and the χ^2^ test, Mann–Whitney U test, and Kruskal–Wallis test examined differences in independent proportions (for parametric and nonparametric distributions, as appropriate). Multivariate logistic regression analysis and generalized linear modeling identified predictors of treatment use and prescribing changes in the total patient sample, including the contribution of CARAT. We adjusted for selected covariates (eg, patient characteristics, including levels of risk) and clustering of patients by GPs according to the level of variability in the initial analyses (eg, inclusion of intercepts for patients nested within GP practices). However, the lack of variability in the control arm of primary outcomes (ie, no changes in treatment use) precluded specific analyses. Simpler analyses focused on treatment changes in the intervention arm (before and after measures). Forward stepwise (Wald) regression was used because of the large number of potential explanatory variables for the outcome. Forward selection of variables begins with no variables in the initial model, then adds variables in succession, with iterative testing to see how each variable addition improves the model. In this study, any variable found to be independently associated with the outcome variable during univariate analysis (*P* < .10) was applied to the multivariate model. The Fleiss κ statistic assessed the level of agreement between the CARAT recommendations and the GP’s final decision on antithrombotic therapy (22–24). All analyses were set at a significance level of .05 (unless otherwise specified), assuming intention-to-treat.

## Results

A total of 393 AFib patients (mean [standard deviation] age, 78.0 [7.0] y) were recruited into the study by 48 GPs; 25 GPs and 206 patients were randomized into the intervention arm (Table 1). We found no significant differences in patient characteristics between the study arms or across the 4 regions, except that patients in the control arm had fewer chronic conditions than patients in the intervention arm (5.4 vs 6.1, *P* = .01).

**Table 1:** Characteristics of Patients With Atrial Fibrillation in Intervention and Control Arms of a Cluster-Randomized Controlled Trial of a Computerized Antithrombotic Risk Assessment Tool for General Practitioners in New South Wales, Australia^a^^,^ b

Characteristic	All Patients	Intervention Arm	Control Arm	*P* Value^c^
**No. of patients (% of total sample)**	393 (100.0)	206 (52.4)	187 (47.6)	—
**Age, mean (SD), y**	78.0 (7.0)	78.2 (7.1)	77.7 (7.0)	.52
**Age group**				
≥80 y	180 (45.8)	96 (46.6)	84 (44.9)	.74
<80 y	213 (54.2)	110 (53.4)	103 (55.1)	
**Sex**				
Male	214 (54.5)	113 (54.9)	101 (54.0)	.87
Female	179 (45.5)	93 (45.1)	86 (46.0)	
**No. of chronic conditions, mean (SD)**	5.8 (2.5)	6.1 (2.7)	5.4 (2.3)	.01
**No. of prescription medications, mean (SD)**	9.2 (4.0)	9.0 (3.7)	9.4 (4.3)	.42
**No. of nonprescription medications, mean (SD)**	1.5 (1.3)	1.5 (1.3)	1.5 (1.3)	.94
**History of atrial fibrillation**				
<3 mo	10 (2.5)	7 (3.4)	3 (1.6)	.24
<12 mo	39 (9.9)	20 (9.7)	19 (10.2)	
<2 y	59 (15.0)	25 (12.1)	34 (18.2)	
<5 y	82 (20.9)	49 (23.8)	33 (17.6)	
≥5 y	203(51.7)	105 (51.0)	98 (52.4)	
**Type of atrial fibrillation**				
Paroxysmal	139 (35.4)	76 (36.9)	63 (33.7)	.86
Persistent	224 (57.0)	116 (56.3)	108 (57.8)	
New onset	22 (5.6)	10 (4.9)	12 (6.4)	
Unknown	8 (2.0)	4 (1.9)	4 (2.1)	
**Duration of persistent atrial fibrillation, mean (SD), y**	6.8 (6.8)	6.0 (3.7)	7.7 (8.9)	.01
**Previous hospitalization for atrial fibrillation**				
No	259 (65.9)	134 (65.0)	125 (66.8)	.71
Yes	134 (34.1)	72 (35.0)	62 (33.2)	
**Reason for previous hospitalization**				
Management of atrial fibrillation	87 (64.9)	48 (66.7)	39 (62.9)	.85
Stroke or cerebrovascular accident from atrial fibrillation	24 (17.9)	13 (18.1)	11 (17.7)	
Transient ischemic attack	12 (9.0)	5 (6.9)	7 (11.3)	
Other^d^	11 (8.2)	6 (8.3)	5 (8.1)	
**Current cardiac rhythm**				
Normal sinus rhythm	45 (11.5)	25 (12.1)	20 (10.7)	.57
Controlled atrial fibrillation	347 (88.3)	180 (87.4)	167 (89.3)	
Uncontrolled atrial fibrillation	1 (0.3)	1 (0.5)	0 (0.0)	

a All values are number (percentage in subgroup) unless otherwise indicated.

b General practitioners were recruited from 4 regions of general practice in New South Wales, Australia: the northern suburbs of Sydney, the Central Coast, the Newcastle metropolitan area (in the Hunter Region), and the rural areas of the Hunter Region. Each general practitioner (25 in control arm and 25 in intervention arm) recruited 5 to 10 patients.

c *P* value for difference between intervention arm and control arm determined by Pearson χ^2^ test.

d Includes acute myocardial infarction, amiodarone-induced hyperthyroidism, anterior resection, aortic valve replacement, carcinoma, cardiac ablation, pacemaker reinsertion, pulmonary embolism.

Overall, 70.7% of patients were assessed as being at high risk of stroke; the most common risk factors were hypertension (68.2%) and being aged 75 or older (67.7%). Only 1.0% of patients were assessed as being at high risk of bleeding; the most common risk factors were uncontrolled hypertension (10.2%) and malignancy (8.4%) (Table 2). A higher proportion of patients in the control arm (11.2%) were deemed to be at low risk of stroke than the proportion in the intervention arm (2.9%) (*P* < .001). Two-thirds (67.9%) of all patients were deemed to be at high risk of stroke *and* at low risk of bleeding (ie, most eligible for antithrombotic therapy).

**Table 2:** Risk Factors and Level of Assessed Risk Among Patients With Atrial Fibrillation in Intervention and Control Arms of a Cluster-Randomized Controlled Trial of a Computerized Antithrombotic Risk Assessment Tool for General Practitioners in New South Wales, Australia^a^

Risk Factor and Level	All Patients (N = 393)	Intervention Arm (n = 206)	Control Arm (n = 187)	*P* Value^b^
**Stroke Risk^c^**				
**Risk factor**				
Previous stroke or transient ischemic attack	72 (18.3)	50 (24.3)	22 (11.8)	.001
Aged ≥75 y	266 (67.7)	151 (73.3)	115 (61.5)	.01
Congestive heart failure	100 (25.4)	84 (40.8)	16 (8.6)	<.001
Hypertension	268 (68.2)	166 (80.6)	102 (54.5)	<.001
Diabetes mellitus	78 (19.8)	41 (19.9)	37 (19.8)	0.98
**Level of assessed risk**				
High	278 (70.7)	175 (85.0)	103 (55.1)	<.001
Intermediate	88 (22.4)	25 (12.1)	63 (33.7)	
Low	27 (6.9)	6 (2.9)	21 (11.2)	
**Bleeding Risk** d				
**Risk factor**				
Hepatic or renal disease	18 (4.6)	12 (5.8)	6 (3.2)	.22
Alcohol abuse	8 (2.0)	7 (3.4)	1 (0.5)	.045
Malignancy	33 (8.4)	22 (10.7)	11 (5.9)	.09
Reduced platelet count	6 (1.5)	6 (2.9)	0 (0.0)	.02
Re-bleeding risk	4 (1.0)	4 (1.9)	0 (0.0)	.06
Uncontrolled hypertension	40 (10.2)	13 (6.3)	27 (14.4)	.008
Anemia	14 (3.6)	9 (4.4)	5 (2.7)	.36
Risk of excessive falls	22 (5.6)	22 (10.7)	0 (0.0)	<.001
Previous hemorrhagic stroke	6 (1.5)	6 (2.9)	0 (0.0)	.02
**Level of assessed risk**				
High	4 (1.0)	4 (1.9)	0 (0.0)	.03
Intermediate	10 (2.5)	8 (3.9)	2 (1.1)	
Low	379 (96.4)	194 (94.2)	185 (98.9)	

a All values are number (percentage in subgroup) unless otherwise indicated.

b *P* value for difference between intervention arm and control arm determined by Pearson χ^2^ test.

c Patients were assessed for risk of stroke according to the CHADS_2_ Score for Atrial Fibrillation Stroke Risk (25).

d Patients were assessed for risk of bleeding according to HEMORR_2_HAGES criteria (26).

We found no significant differences between the 2 arms in factors affecting the decision to prescribe antithrombotic therapy (Table 3). Overall, the most common medication-safety considerations were patients taking 4 or more medications (94.4%), patients needing assistance with medication management (41.0%), and functional impairment (16.0%). Although most patients were taking 4 or more medications, only 5.6% had a known history of medication nonadherence. Most patients (96.7%) had been educated about their antithrombotic therapy; only 1.3% of patients had previously declined therapy.

**Table 3:** Medication-Safety and Medication-Management Assessments in the Intervention and Control Arms of a Cluster-Randomized Controlled Trial of a Computerized Antithrombotic Risk Assessment Tool for General Practitioners in New South Wales, Australia^a^

Assessment	All Patients (N = 393)	Intervention Arm (n = 206)	Control Arm (n = 187)	*P* Value^b^
**Medication safety**				
Patient allergic to warfarin and aspirin	8 (2.0)	6 (2.9)	2 (1.1)	.22
Adverse reaction to antithrombotics	15 (3.8)	9 (4.4)	6 (3.2)	.52
Taking medication that interacts with warfarin	1 (0.3)	1 (0.5)	0 (0.0)	.73
Patient has declined antithrombotics	5 (1.3)	4 (1.9)	1 (0.5)	.38
Patient has contraindication to antithrombotics	11 (2.8)	5 (2.4)	6 (3.2)	.76
Patient has failed antithrombotics	10 (2.5)	6 (2.9)	4 (2.1)	.75
Patient received education about antithrombotics	380 (96.7)	198 (50.4)	182 (47.9)	.58
**Medication management**				
Patient taking ≥4 medications	371 (94.4)	195 (94.7)	176 (94.1)	.83
Patient is not compliant with medication	22 (5.6)	8 (3.9)	14 (7.5)	.13
Patient needs assistance for medication management	161 (41.0)	83 (40.3)	78 (41.7)	.84
Difficulty accessing medical care	3 (0.8)	1 (0.5)	2 (1.1)	.61
Patient in residential care facility	4 (1.0)	2 (1.0)	2 (1.1)	>.99
Cognitive impairment	18 (4.6)	8 (3.9)	10 (5.3)	.63
Vision impairment	24 (6.1)	15 (7.3)	9 (4.8)	.40
Hearing impairment	34 (8.7)	18 (8.7)	16 (8.6)	>.99
Language/communication barrier	4 (1.0)	2 (1.0)	2 (1.1)	>.99
Mobility disorder	17 (4.3)	9 (4.4)	8 (4.3)	>.99
Functional impairment	63 (16.0)	26 (12.6)	37 (19.8)	.06

a All values are number (percentage in subgroup) unless otherwise indicated.

b *P* value for difference between intervention arm and control arm determined by Pearson χ^2^ test.

**Figure 2:**
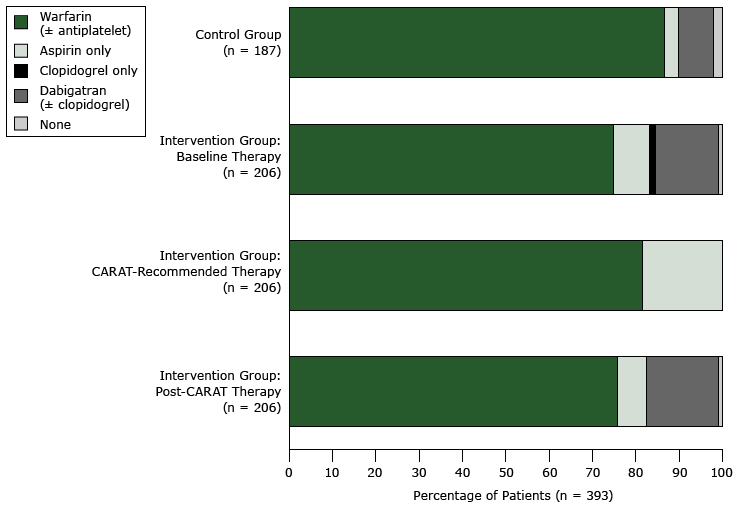
Changes in the use of antithrombotic therapy, by type of therapy and by patient groups (intervention arm and control arm), in a cluster-randomized controlled trial of a computerized antithrombotic risk assessment tool in a sample of general practices in New South Wales, Australia, 2012–2013. Percentages may not total 100 because of rounding. **Table:** 

At baseline, 387 patients (98.5%) used some form of antithrombotic therapy: 361 (91.9%) used an anticoagulant (warfarin [± antiplatelet] or dabigatran [± clopidogrel]), 26 (6.6%) used antiplatelet therapy (aspirin only or clopidogrel only), and 6 (1.5%) did not use any therapy (Figure 2). Most patients using antithrombotics were prescribed warfarin (316/387; 81.7%); of these, 14 used warfarin in combination with aspirin, and 1 patient each was prescribed the combination of warfarin, aspirin, and clopidogrel or warfarin and clopidogrel. The remaining patients were prescribed dabigatran (± clopidogrel) (45/387; 11.6%), aspirin only (23/387; 5.9%), or clopidogrel only (3/387; 0.8%).

Therapy	Number of Patients (Proportion of Patients by Intervention Group and Control Group)			
	Intervention Group: Baseline Therapy	Intervention Group: Post-CARAT Therapy	Intervention Group: CARAT-Recommended Therapy	Control Group
Warfarin (± antiplatelet)	154 (74.8)	156 (75.7)	168 (81.6)	162 (86.6)
Aspirin only	17 (8.3)	14 (6.8)	38 (18.4)	6 (3.2)
Clopidogrel only	3 (1.5)	0 (0.0)	0 (0.0)	0 (0.0)
Dabigatran (± clopidogrel)	30 (14.6)	34 (16.5)	0 (0.0)	15 (8.0)
None	2 (1.0)	2 (1.0)	0 (0.0)	4 (2.1)
Total	206 (100.2)	206 (100.0)	206 (100.0)	187 (99.9)

At baseline, the use of anticoagulants was similar between patients in the control arm (177/187; 94.7%) and intervention arm (184/206; 89.3%) (*P* = .38) as was the use antiplatelet therapy (6/187 [3.2%] in control arm vs 20/206 [9.7%] in intervention arm; *P* = .10). Although the difference was not significant, a greater proportion of patients in the control arm (162/187; 86.6%) used warfarin (± antiplatelet) than in the intervention arm (154/206; 74.8%), and a smaller proportion in the control arm (15/187; 8.0%) used dabigatran (± clopidogrel) than in the intervention arm (30/206; 14.6%).

In the intervention arm, all 206 patients were recommended some form of antithrombotic therapy by CARAT; no patient was recommended no therapy. Overall, CARAT recommended a different type of therapy or agent for 75 (36.4%) patients; for 1 other patient, the change was to increase the dose of warfarin to achieve therapeutic levels at the recommended target international normalized ratio. Among the 75 CARAT-recommended changes, 12 (16.0%) were upgrades to a more effective prophylactic therapy (ie, from no therapy to any agent, or from aspirin to warfarin or dabigatran), whereas 35 (46.7%) were classified as “side-steps” to different agents in the same category of therapy (eg, from one anticoagulant to another, or from the combination of warfarin and aspirin to warfarin only). The remaining 28 (37.3%) recommendations were downgrades to less effective but potentially safer options (ie, from any agent to no therapy, or from warfarin and dabigatran to aspirin). Among the 6 patients using the combination of warfarin and aspirin for whom CARAT recommended changes, CARAT recommended changing the therapy to warfarin only for all but one patient (who was recommended aspirin only).

GPs agreed with most (75.2%) CARAT recommendations for the 206 patients in the intervention arm (κ = 0.91; *P* = .03). Overall, for the 75 recommended changes, GPs agreed with the type of therapy (ie, antiplatelet vs anticoagulant) suggested by CARAT in 48 (64.0%) cases. However, among these 48 cases, GPs disagreed with CARAT on the agent recommended in 30 (62.5%) cases, all relating to the GP preferences for dabigatran over the CARAT-recommended warfarin. Among the 35 recommendations for warfarin-only therapy, GPs agreed in 13 cases; most GPs instead prescribed dabigatran (19 cases) followed by aspirin (2 cases), and 1 patient received no therapy. The most common reasons cited for disagreeing with CARAT’s recommendations were “treatment considered inappropriate” and “following the cardiologist’s recommendation.” In the northern suburbs of Sydney, the main reason for declining CARAT’s recommendation was participation in the Pradaxa (dabigatran) product familiarization program, regardless of whether the NOAC was appropriately indicated or not. Perceived patient refusal was cited as a reason to decline CARAT recommendations in 8 of 48 (16%) intervention patients (ranging from 6.7% to 33.3% across practice regions); however, according to clinical notes, only 4 (1.9%) intervention patients declined therapy previously (Table 3).

GPs initiated changes to therapy only among intervention patients flagged by CARAT as needing a change; GPs did not make any changes to therapy for the 131 patients for whom CARAT did not recommend a change. Overall, the net effect of these changes was that the use of antithrombotic therapy did not change from baseline (204/206; 99.0%), but the use of anticoagulants increased significantly (*P* = .02) from 89.3% (184/206) to 92.2% (190/206), and the use of antiplatelet therapy decreased (but not significantly [*P* = .20]) from 9.7% (20/206) to 6.8% (14/206) (Figure 2). None of the 3 patients using clopidogrel at baseline used it after application of CARAT: 2 patients were prescribed dabigatran and 1 was prescribed warfarin and aspirin. These changes are net changes in the proportion of patients using these antithrombotics, taking into account all upgrades, downgrades, and side-steps in treatment. We found more CARAT recommendations for downgrades to less effective but potentially safer options than for upgrades to more effective prophylactic therapy.

Overall, a significantly higher proportion of patients in the intervention arm (36.9%; 76/206) than in the control arm (0%; 0/187) were assessed for potential changes to therapy during baseline consultations (*P* < .001); 9 (4.4%; 9/206) patients in the intervention arm and none in the control arm immediately changed their therapy (*P* = .003).

Overall, compared with patients in the control arm, patients in the intervention arm were 3.1 times more likely to be considered for warfarin therapy (in preference to other treatment options; *P* < .001) and 2.8 times more likely to receive an anticoagulant (in preference to an antiplatelet; *P* = .02) (Table 4).

**Table 4:** Predictors of the Use of Antithrombotic Therapy (Multivariate Model) Post-Intervention (N = 393) in a Cluster-Randomized Controlled Trial of a Computerized Antithrombotic Risk Assessment Tool (CARAT) for General Practitioners in New South Wales, Australia

Variable	Odds Ratio (95% Confidence Interval)
**Model A: Predictors of the use of anticoagulant therapy in preference to antiplatelet therapy^a^**	
Use of CARAT for decision making in the intervention arm (vs control arm)	2.8 (1.1–7.3)^b^
History of uncontrolled hypertension	3.5 (1.4–8.3)^b^
Previous hemorrhagic stroke	0.1 (0.02–0.7)
**Model B: Predictors of the use of warfarin in preference to other treatment options^c^**	
Intervention arm decision making after application of CARAT (vs control arm)	3.1 (1.7–5.7)^b^
History of uncontrolled hypertension	2.4 (1.3–4.1)^b^
Excessive alcohol intake	0.2 (0.05–1.0)
Language barrier	0.1 (0.01–0.7)
Increasing number of prescription medications being used	0.8 (0.6–0.9)
Increasing number of nonprescription medications being used	0.8 (0.6–1.0)
Malignancy	0.4 (0.2–0.8)
Previous hemorrhagic stroke	0.2 (0.3–0.97)

a Model correctly classified 93.5% of cases. Cox and Snell *R* ^2^ = 0.43; Nagelkerke *R* ^2^ = 0.11.

b More likely to receive the former therapy; analyses adjusted for selected factors.

c Model correctly classified 81.4% of cases. Cox and Snell *R* ^2^ = 0.12; Nagelkerke *R* ^2^ = 0.19.

## Discussion

GPs in local Australian practices are increasingly prescribing antithrombotic therapy for stroke prevention in patients with AFib, in line with current guidelines and in response to interventions implemented during the past decade to improve clinician awareness, better support patients and clinicians in managing anticoagulant therapy, and develop newer treatment options. Overall, the high use of antithrombotics (any therapy) in this cohort of patients demonstrates that tools such as CARAT may have a limited role in the initial decision to prescribe therapy; however, the choice of agents may require further support, as evidenced by the proportion of patients receiving antiplatelet therapy or dabigatran, and which may reflect persistent reservations about warfarin use. This is an interesting finding, given that the prevalence of contraindications to warfarin and any related medication safety considerations that are often cited as reasons to avoid warfarin (eg, falls risk, functional/cognitive impairment) was low in this study. Most patients were assessed as being at high risk of stroke and at low risk of bleeding and therefore were ideal candidates for anticoagulation therapy with warfarin. More important was that CARAT helped to rationalize therapy rather than simply increasing therapy: the tool helped to review a patient’s existing therapy and accounted for any changes in risk (for both stroke and bleeding) and development of contraindications; this process resulted in more downgrades (to safer options) than upgrades (to more effective options) in therapy. Studies have similarly shown this effect when review processes and tools are not only applied in the initial decision to prescribe therapy but also in reviewing a patient’s ongoing therapy (14).

Although the application of CARAT did not result in dramatic improvements in the net use of antithrombotic therapy in this study, because of the high use of anticoagulants at baseline, the level of agreement on recommendations between CARAT and GPs is encouraging. However, the GPs agreeing to participate in this study may have been more confident in their evidence-based practice in antithrombotic therapy than GPs not agreeing to participate, and this potential selection bias may have contributed to the unexpectedly high level of concordance with antithrombotic guidelines. Nevertheless, CARAT assisted with rationalization of therapy and identified several lingering issues, such as patient refusal, that appear to preclude further improvement in care; future research should explore how adherence to therapy can be better supported and how clinicians can better engage patients in the decision-making process.

Since this study was conducted, the NOACs (dabigatran, rivaroxaban, apixaban) have become available for widespread use (at the Australian Pharmaceutical Benefits Scheme’s subsidized costs) and have complicated decision-making further, necessitating guidance on how to select a therapy (5,7). Although these new agents offer several advantages, each has risks; safety alerts have described the risks of over-zealous, indiscriminate use (27). In this study, numerous patients who were recommended warfarin by CARAT received dabigatran instead, with no obvious rationale for dabigatran’s selection. Dedicated tools may be needed to help clinicians identify patients who would benefit from NOAC therapy and to select which type to use; for this reason, a modified version of the CARAT is in development. How the high costs of NOACs may affect prescribing patterns should be considered. Although NOACs were found to be cost-effective alternatives to warfarin for stroke prevention in patients with AFib (28), studies showed that the absolute, short-term costs of NOACs can affect treatment selection. For example, a Canadian study demonstrated socioeconomic inequality in access to dabigatran among patients receiving warfarin for nonvalvular AFib; in the absence of subsidies, patients in the highest income quintile were 50% more likely to switch from warfarin to dabigatran than those in the lowest income quintile (29). When such disparity is eliminated through subsidies, treatment costs challenge health-system budgets. In Australia, high-cost medicines such as NOACs are subsidized by the Australian Pharmaceutical Benefit Scheme, making them affordable for patients but costly for government. Concerns about the costs of NOACs led the Australian government to re-examine the selection of therapy (7). In this study, prescribing patterns were likely not affected by the differential costs of treatments because of subsidies. However, decision-support tools such as CARAT may need to consider how costs are weighed in the decision-making processes of the diverse economic and health-systems contexts in which therapies are used.

This study had several limitations. First, data extraction relied on GP responses and clinical records; records may have been outdated or incomplete, and explanations for the use of therapy may not have been recorded. In particular, the patient’s AFib status was determined by GP verification; however, the proportion of patients with new onset AFib (in whom the need for long-term antithrombotic therapy may not initially be clear) was low (5.6%) and had no significant effect on overall treatment use. Second, routine anticoagulation therapy may not be indicated for all cases of new onset AFib, such as post-surgical AFib, and so the role of CARAT may be limited in this context (30). Third, this study was designed to explore the use of therapy according to prescription by clinicians; it did not examine the extent to which patients adhered to the treatments prescribed.

CARAT was designed only to consider antithrombotics available at the time of the study (ie, warfarin, aspirin), and it may not have accounted for other factors important to decision-making, such as patients preferences (eg, warfarin vs NOAC), affordability, and access to resources to support clinicians and patients in managing treatment options. The availability of dabigatran through the product familiarization program may have clouded decision making in some cases, and the full impact of CARAT may not have been realized. The availability of dabigatran through the product familiarization program may have motivated some clinicians to try it, regardless of CARAT’s recommendation. Concerns about the product familiarization program and possible inappropriate use of dabigatran have been raised (31). The inability of CARAT to recommend a NOAC as a treatment option or provide practical advice to clinicians about the suitability of dabigatran for a patient may have affected the rate of uptake of CARAT recommendations. A higher proportion of control-arm patients than intervention-arm patients were deemed to be at low risk of stroke; overall, this difference did not appear to influence the likelihood of receiving treatment, according to the multivariate analyses. Collectively, the above-mentioned issues may have affected the impact of CARAT and the patterns of antithrombotic use observed in this study.

Despite these limitations, this study showed that CARAT improved the use of antithrombotic therapy by supporting the rationalization of therapy. Tools such as CARAT support decision making by assisting in selecting a therapy, particularly in upgrading a patient from an antiplatelet agent to an anticoagulant. The advent of NOACs has further complicated decision making, and decision-support tools must evolve to weigh the risks and benefits of these new therapy options.
